# NOD2 promotes dopaminergic degeneration regulated by NADPH oxidase 2 in 6-hydroxydopamine model of Parkinson’s disease

**DOI:** 10.1186/s12974-018-1289-z

**Published:** 2018-08-29

**Authors:** Li Cheng, Lin Chen, Xinbing Wei, Yimeng Wang, Zhiping Ren, Shenglan Zeng, Xiumei Zhang, Haitao Wen, Chengjiang Gao, Huiqing Liu

**Affiliations:** 1 Department of Pharmacology, School of Basic Medical Sciences, Shandong University, Wenhua West Road 44, Jinan, 250012 Shandong People’s Republic of China; 2 Department of Microbial Infection and Immunity, Infectious Disease Institute, The Ohio State University, Columbus, OH USA; 3 State Key Laboratory of Microbial Technology, Key Laboratory of Infection and Immunity of Shandong Province, Department of Immunology, School of Basic Medical Sciences, Shandong University, Jinan, 250012 Shandong China

**Keywords:** Nucleotide-binding oligomerization domain-containing protein 2 (NOD2), Neuroinflammation, Microglia, Cytokine, Apoptosis, Reactive oxygen species (ROS), NADPH oxidase 2 (NOX2), Parkinson’s disease

## Abstract

**Background:**

In Parkinson’s disease (PD), loss of striatal dopaminergic (DA) terminals and degeneration of DA neurons in the substantia nigra (SN) are associated with inflammation. Nucleotide-binding oligomerization domain-containing protein (NOD)2, one of the first discovered NOD-like receptors, plays an important role in inflammation. However, the role of NOD2 has not been elucidated in PD.

**Methods:**

NOD2 mRNA and protein expression in the SN and the striatum of C57BL/6 mice treated with 6-hydroxydopamine (6-OHDA) was measured. We next investigated the potential contribution of the NOD2-dependent pathway to 6-OHDA-induced DA degeneration using NOD2-deficient (NOD2^−/−^) mice. Assays examining DA degeneration and inflammation include HPLC, Western blot, immunohistochemistry, TUNEL staining, and cytometric bead array. To further explore a possible link between NADPH oxidase 2 (NOX2) and NOD2 signaling in PD, microglia were transfected with shRNA specific to NOX2 in vitro and apocynin were given to mice subjected to 6-OHDA and muramyl dipeptide (MDP) striatal injection.

**Results:**

The expression of NOD2 was upregulated in an experimental PD model induced by the neurotoxin 6-OHDA. NOD2 deficiency resulted in a protective effect against 6-OHDA-induced DA degeneration and neuronal death, which was associated with the attenuated inflammatory response. Moreover, silencing of NOX2 in microglia suppressed the expression of NOD2 and the inflammatory response induced by 6-OHDA and attenuated the toxicity of conditioned medium from 6-OHDA or MDP-stimulated microglia to neuronal cells. Furthermore, apocynin treatment inhibited NOD2 upregulation and DA degeneration in the SN of WT mice induced by 6-OHDA and MDP.

**Conclusion:**

This study provides the direct evidence that NOD2 is related to 6-OHDA-induced DA degeneration through NOX2-mediated oxidative stress, indicating NOD2 is a novel innate immune signaling molecule participating in PD inflammatory response.

**Electronic supplementary material:**

The online version of this article (10.1186/s12974-018-1289-z) contains supplementary material, which is available to authorized users.

## Background

Parkinson’s disease (PD) is the second most common neurodegenerative disorder characterized by a massive and preferential loss of dopaminergic (DA) neurons in the substantia nigra (SN) and a drastic decline in striatal dopamine concentrations. It is established that sustained neuroinflammation has been suggested to contribute to the pathogenesis of PD [1, 2]. Activated microglia and astrocytes, together with elevated levels of inflammatory mediators and cytotoxic factors including tumor necrosis factor alpha (TNFα); interleukin (IL)-1β, IL-2, and IL-6; inducible nitric oxide synthase (iNOS), and cyclooxygenase-2 (COX2), have all been observed in the brain of PD patients [3–6], as well as in the 6-hydroxydopamine (6-OHDA) [7–10], 1-methyl-4-phenyl-1,2,3,6-tetrahydropyridine (MPTP) [11, 12] and rotenone animal models of PD [13]. However, the mechanisms underlying neuroinflammation currently remain unclear.

Accumulating evidence suggests that activation of innate immunity via Toll-like receptors (TLRs) [13–19] and nucleotide-binding oligomerization domain-containing protein (NOD)-like receptors (NLRs) [20, 21] has been implied to participate in the pathogenesis of PD. NOD2, one of the first discovered NLRs, plays an important role in regulating inflammatory homeostasis. It recognizes not only the exogenous pathogen-associated molecular patterns (PAMPs) such as muramyl dipeptide (MDP), a degradation product of peptidoglycans from virtually all bacteria [22], but also endogenous danger-associated molecular patterns (DAMPs) which are released from the damaged tissues following cellular stress. When engaged by its ligands, NOD2 recruits kinase receptor-interacting serine/threonine-protein kinase 2 (RIP2), leading to the activation of NF-κB and MAPK signaling pathways [23]. It has been reported that mutations in the NOD2 gene are related to inflammatory diseases such as Crohn’s disease, Blau syndrome, and early-onset sarcoidosis [24–27]. However, the data on such mutations in PD are inconclusive [28–30]. This study was designed to elucidate the function of NOD2 signaling and its regulatory mechanism in the pathogenesis of PD.

## Methods

### Animals

Ten-week-old male C57BL/6J mice (weight 23–28 g) were obtained from the Experimental Animal Center of Shandong University. NOD2 knockout (NOD2^−/−^) mice on a C57BL/6 background were purchased from the Jackson Laboratory (Bar Harbor, ME, USA). All mice were housed under standard conditions of temperature and humidity, with a 12-h light/dark cycle and free access to food and water. All animal experiments were pre-approved by the Institutional Animal Care and Use Committee of Shandong University.

### Striatal injections

Mice were placed in a stereotaxic device under 1.5% pentobarbital sodium anesthesia and given 2 μl of 3 μg/μl 6-OHDA (Sigma-Aldrich, H4381) which is dissolved in sterile normal saline (NS) with 0.02% ascorbic acid or 4 μg/μl MDP (an agonist of NOD2, Sigma-Aldrich, A9519) solution into two different sites of the right striatum (STR) separately: point A—1.0 mm anterior and 2.1 mm lateral to the bregma and 2.9 mm from the dura mater; point B—0.3 mm posterior and 2.3 mm lateral to the bregma and 2.9 mm from the dura mater. The injection was conducted at a rate of 0.5 μl/min, and the needle was left in place for an additional 4 min before it was slowly removed. The control group was injected with NS alone (containing 0.2% ascorbic acid) into the STR.

### Apocynin treatment

To evaluate the role of NADPH oxidase in NOD2 signaling in PD, mice were given saline or apocynin (15 mg/kg), a NADPH oxidase inhibitor, by daily intraperitoneal injection for 14 days after 6-OHDA and MDP administration.

### Apomorphine-induced rotation test

Rotation testing was performed according to a previously published protocol [31]. One day, 2 days, 3 days, 7 days, 14 days, and 21 days after 6-OHDA injection, the mice were injected subcutaneously with 0.1 mg/kg apomorphine hydrochloride (Sigma-Aldrich, A4393). Then, the mice were placed individually in a transparent plastic cylinder (diameter 13 cm) and allowed to adapt to their environment for 5 min before the rotations were recorded. The total number of full 360° rotations in the contralateral direction was counted for 30 min. Results were expressed as average rotations per 10 min in 30-min measurement.

### RNA extraction and RT-PCR

RNA extraction and reverse transcription were performed as described previously [32]. The quantitative real-time PCR analysis was performed with SYBR green (Invitrogen) using a Bio-Rad iCycler system (Bio-Rad). Housekeeping gene expression of β-actin was used for normalization. Relative expression was determined by the 2^−ΔΔCT^ method. The specific primers used were β-actin (sense 5′-GAATTGCTATGTGTCTGGGT-3′, antisense 5′-CATCTTCAAACCTCCATGATG-3′) and NOD2 (sense5′-GCCTTCCTTCTACAGCACGT-3′, antisense 5′-TGGCAGGGCTCTTCTGCAAG-3′).

### Immunoblotting

Proteins were prepared as previously described [33]. The membranes were incubated with the following primary antibodies: TH (Millipore, MAB318, 1:2000), NOD2 (Proteintech, 20980, 1:1000; Group), NOX2 (BD, 611414, 1:1000), Bax (Proteintech, 50599, 1:2000), Bcl-2 (Proteintech, 12789, 1:1000), cytochrome C (Proteintech, 10993, 1:1000), cleaved caspase-3 (Bioworld, BS7004, 1:500), pro caspase-3(Bioworld, BS9865, 1:1000), COX2 (Proteintech, 12375, 1:500), iNOS (Proteintech, 18985, 1:500), NF-κB p-p65 (Cell signaling, 3033, 1:500), NF-κB p65 (Proteintech, 10745, 1:1000), IκBα (Bioworld, BS3601, 1:1000), or β-Actin (Bioworld, AP0060, 1:5000). The membranes were then incubated with HRP-conjugated secondary antibodies for 1 h. Subsequent visualization was performed using an enhanced chemiluminescence system (ECL, Millipore). Densitometry of Western blot bands was assessed with the image lab program and then normalized to the intensity of β-actin.

### Immunohistochemistry

Mice were anesthetized with sodium pentobarbital 14 days after 6-OHDA or MDP administration, and the brains were perfusion-fixed with 4% paraformaldehyde (PFA) following 0.9% NS. Then, the brains were post-fixed in 4% PFA for 24 h and cryopreserved in 30% sucrose in turns. The brain sections (20 μm or 10 μm) were obtained on a sliding microtome adapted for cryosectioning. The sections were incubated with the following primary antibodies: NOD2 (Proteintech, 20980, 1:200), TH (Millipore, MAB318, 1:200), Iba1 (Proteintech, 10904, 1:200), glial fibrillary acidic protein (GFAP) (Millipore, MAB360, 1:200), and Neun (Millipore, MAB377, 1:200). For immunofluorescence staining, the sections were counterstained with DAPI. The morphometric analyses were performed using the program Image J (NIH, Bethesda, MA).

### High-performance liquid chromatography-tandem mass spectrometry

Dopamine and its metabolites, dihydroxyphenylacetic acid (DOPAC), and homovanillic acid (HVA) were measured using HPLC/MS method. Briefly, the right striatum was individually weighed and homogenized in ice-cold 0.5 M formic acid with the concentration of 5 ml/g tissue. Lysates were centrifuged at 15,000*g* for 30 min at 4 °C. The supernatant was separated and analyzed according to the established protocols with minor modifications [34]. The concentration was expressed as nanogram per milligram tissue.

### TUNEL staining

TUNEL staining was performed using an in situ apoptosis detection kit (Roche, 11684817910) according to the manufacturer’s instructions. TUNEL-positive cells displayed brown staining within the nucleus, and the number of TUNEL-positive cells was counted in three non-overlapping microscopic eyeshots by a person blinded to the group assignment under high-power magnification (× 200) and displayed as a percentage.

### Cytometric bead array assay

IL-6, IL-12p70, MCP-1, TNFα, and IL-10 in mice SN were captured by cytometric bead array (BD, 552364) according to the manufacturer’s manual. Cytokine levels were then quantified by flow cytometry (Beckman Coulter).

### ROS measurement

ROS generation in the SN or microglia was measured by the fluorescence intensity of dichlorofluorescein (DCF) converted from 2′,7′-dichlorofluorescein diacetate (DCFH-DA) at 525 nm after excitation at 488 nm by a fluorescence plate reader (Thermo Scientific Varioskan Flash).

### Cell culture and treatment

Microglia BV2 cells were cultured in Dulbecco’s modified Eagle’s medium (DMEM; Gibco) supplemented with 10% fetal bovine serum (FBS; Gibco). The neurotoxin 6-OHDA was dissolved in 0.02% ascorbic acid and prepared fresh for each experiment. Cultures were exposed to 50 μM 6-OHDA for indicated time before being harvested for various assays. shRNA-NOX2 was synthesized by GenePharma Co., Ltd. (Shanghai, China). The target sequence for shRNA-NOX2 (5′-GAGTGGTGTGTGAATGCCAGA-3′) was designed based on the core sequence of mouse NOX2 cDNA (accession number: NM_007807.4). Transfection was performed using lipofectamine 3000 reagent (Invitrogen).

The human neuroblastoma cell line SH-SY5Y cells were maintained in DMEM-F12 (Hyclone) containing 10% FBS. Conditioned medium (CM) from 6-OHDA or MDP-treated BV2 cells was collected from wells, pooled, and centrifuged at 170*g* for 5 min to remove cell debris. SH-SY5Y cells were cultured in 96-well plates at 10,000 cells/well and were incubated for 24 h before the addition of BV2 CM. The original media was removed from SH-SY5Y cell cultures and replaced with 100 μl of DMEM-F12 mixed with 100 μl of BV2 CM. The SH-SY5Y cells were then incubated for 24 h. Cell viability was determined by the Cell Counting Kit-8 (CCK-8) (Beyotime, C0038) assay.

### Statistical analysis

Data analyses were performed using the SPSS statistical software. Statistical significance between multiple groups was analyzed by one-way ANOVA followed by LSD post hoc test. When equal variances were not assumed, Dunnett’s multiple comparisons test was used to compare the differences between the groups. Comparison of the two groups was performed using two-tailed *t* test. All data are presented as mean ± SEM, and *p* < 0.05 was considered a statistically significant difference.

## Results

### Increased NOD2 expression in the 6-OHDA-induced PD mouse model

**Fig. 1:**
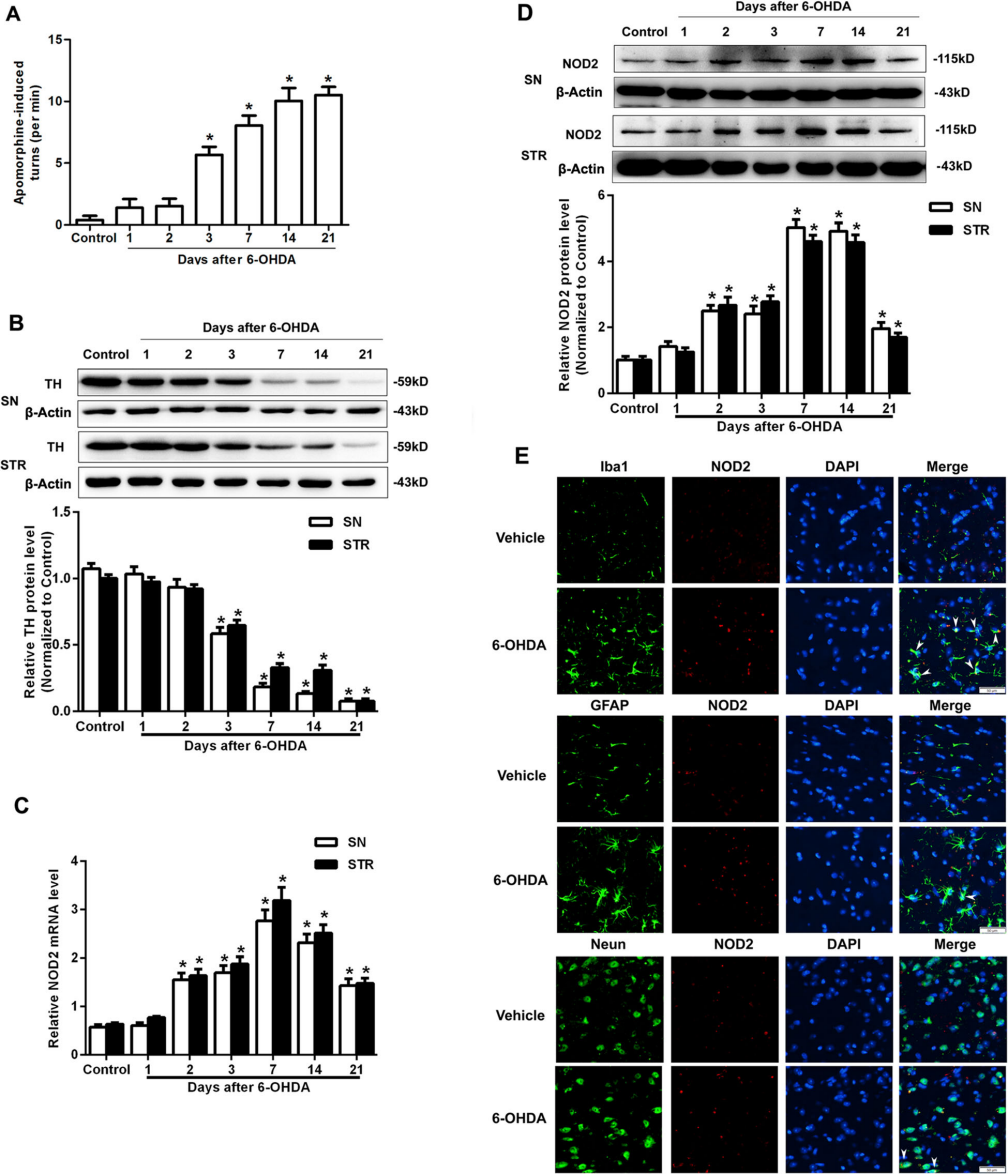
The expressions of NOD2 in the substantia nigra (SN) and striatum (STR) were increased in Parkinson’s disease mice induced by 6-OHDA. Wild-type mice were placed in a stereotaxic device under 1.5% pentobarbital sodium anesthesia and given 6-OHDA or saline alone (control) into the STR. **a** Apomorphine-induced circling on 1 day, 2 days, 3 days, 7 days, 14 days, and 21 days after the injection was shown as the average rotations per 10 min in 30 min after subcutaneous injection of apomorphine. **b** Western blot analysis showed the protein expression of tyrosine hydroxylase (TH) in mouse SN and STR after 6-OHDA injection respectively. **c** Relative quantitative mRNA levels of NOD2 in mouse SN and STR after 6-OHDA injection were determined by RT-PCR analysis. **d** Western blot analysis showed the protein expression of NOD2 in mouse SN and STR after 6-OHDA injection. All data are presented as the mean ± SEM, *n* = 6 mice per group. *p < 0.05 vs. control group. **e** Representative images of double immunolabeling for NOD2 and Iba1 (microglia marker), glial fibrillary acidic protein antibody (GFAP, astrocyte marker), or NeuN antibody (neuron marker) in the SN from WT mice on 14 days after 6-OHDA treatment. DAPI indicates 4′,6-diamidino-2-phenylindole. Arrowheads show NOD2-positive cells. Scale bars, 50 μm

In our study, 6-OHDA-induced DA neuronal lesion in mice was used as an animal model of PD. Three days to 21 days after 6-OHDA injection, apomorphine stimulated obvious contralateral rotation and Western blot showed a time-dependent reduction of TH, a specific enzyme expressed in DA neurons, both in the SN and STR. These results indicated 6-OHDA injection in the STR induced a significant depletion of dopamine (Fig. 1a, b). We also observed an increase of the mRNA and protein levels of NOD2 in the SN and STR starting from 2 days after the lesion in WT mice. This increase reached the peak at 7 days and persisted till to 14 days after the lesion (Fig. 1c, d). The expression and cellular localization of NOD2 were further confirmed by double immunofluorescence staining. NOD2 was increased in the SN from WT mice at 14 days after 6-OHDA injection and mainly present in microglia (Fig. 1e).

### The striatal injection of the NOD2 agonist MDP-induced dopaminergic degeneration

**Fig. 2:**
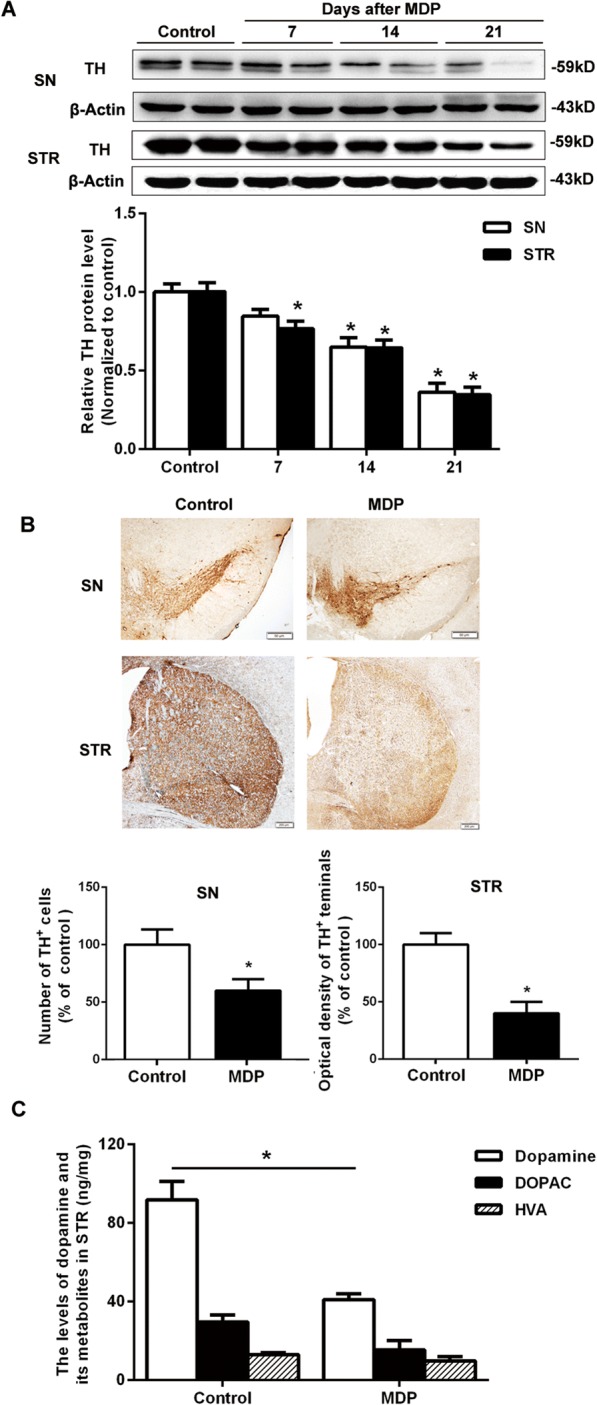
NOD2 stimulation induced dopaminergic degeneration. Wild-type (WT) mice were placed in a stereotaxic device under 1.5% pentobarbital sodium anesthesia and given muramyl dipeptide (MDP, an extrinsic ligand of NOD2) or saline alone into two different sites of the STR separately. **a** Western blot showed the time-dependent reduction of tyrosine hydroxylase (TH) in WT mouse SN and STR after striatal injection of MDP. **b** The brain sections from the SN and STR showing TH immunoreactivity on 14 days after striatal infusion of MDP. **c** The levels of dopamine and its metabolites in the striatum were measured by HPLC/MS analysis on 14 days after MDP injection. All data are presented as the mean ± SEM, *n* = 6 mice per group. *p < 0.05, compared with indicated group

To determine whether NOD2 stimulation leads to the damage of DA neurons, we injected the NOD2 agonist MDP into the STR of adult WT mice. Western blot showed a time-dependent reduction of TH in the SN and STR of WT mice (Fig. 2a) after striatal injection of MDP. Immunohistochemical staining showed that the TH-positive neurons in the SN and TH-positive area in the STR were significantly decreased in WT mice with MDP injection as compared with the NS injection (Fig. 2b). Meanwhile, we also observed that MDP injection led to a significant decrease of dopamine level in the STR of WT mice as compared with the control group (Fig. 2c).

### NOD2 deficiency alleviated dopaminergic degeneration induced by 6-OHDA

**Fig. 3:**
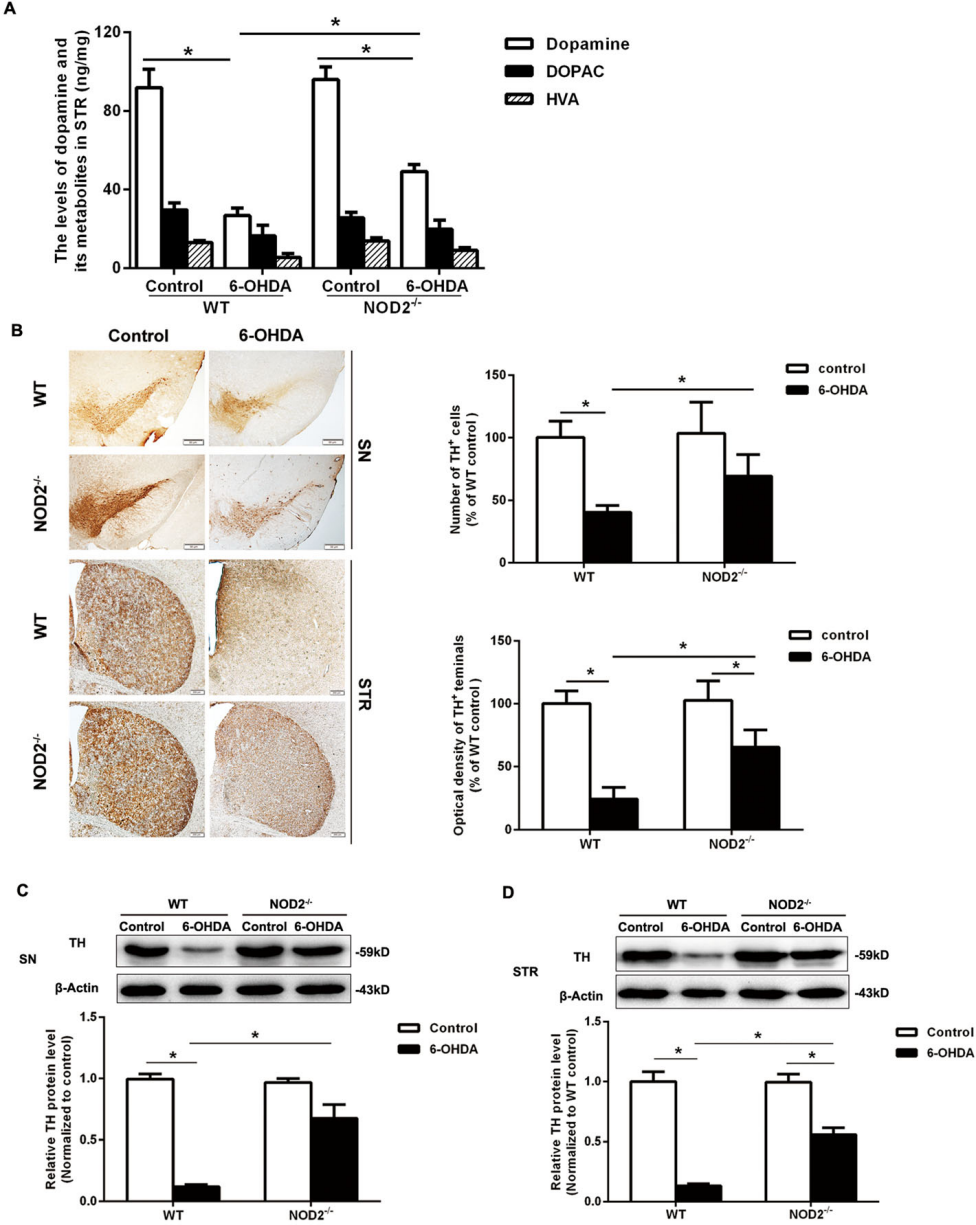
NOD2 deficiency attenuated dopaminergic degeneration induced by 6-OHDA. Wild-type (WT) and NOD2-deficient (NOD2^−/−^) mice were placed in a stereotaxic device under 1.5% pentobarbital sodium anesthesia and given 6-OHDA or saline alone into two different sites of the STR separately. **a** The levels of dopamine and its metabolites in the striatum were measured by HPLC/MS analysis on 14 days after 6-OHDA injection. **b** The brain sections from the SN and STR showing TH immunoreactivity on 14 days after striatal infusion of 6-OHDA. NOD2 deficiency improved the reduction of tyrosine hydroxylase (TH) protein expression in the SN (**c**) and STR (**d**) on 14 days after 6-OHDA injection. All data are presented as the mean ± SEM, *n* = 6 mice per group. *p < 0.05, compared with indicated group

To further evaluate the exact function of NOD2 in PD, NOD2^−/−^ mice were injected 6-OHDA into the STR. As shown in Fig. 3a, WT mice subjected to striatal 6-OHDA injection had a 71.0% decrease in dopamine content as compared to the WT NS-treated group, while NOD2^−/−^ mice exhibited a significantly higher striatal dopamine level than WT mice lesioned by 6-OHDA. TH immunolabeling indicated that striatal 6-OHDA injections in WT mice reduced the number of DA neurons in the SN and TH-positive area in the STR in comparison to the NS group. In contrast, the DA degeneration caused by 6-OHDA in NOD2^−/−^ mice was markedly alleviated compared to WT mice (Fig. 3b). Western blot results also showed NOD2 deficiency attenuated the reduction of TH protein expression in the SN and STR on 14 days after 6-OHDA injection (Fig. 3c, d). All the results further supported that NOD2 play a role in the degeneration of DA neurons in PD.

### NOD2 deficiency attenuated the DA neuronal apoptosis in the SN induced by 6-OHDA

**Fig. 4:**
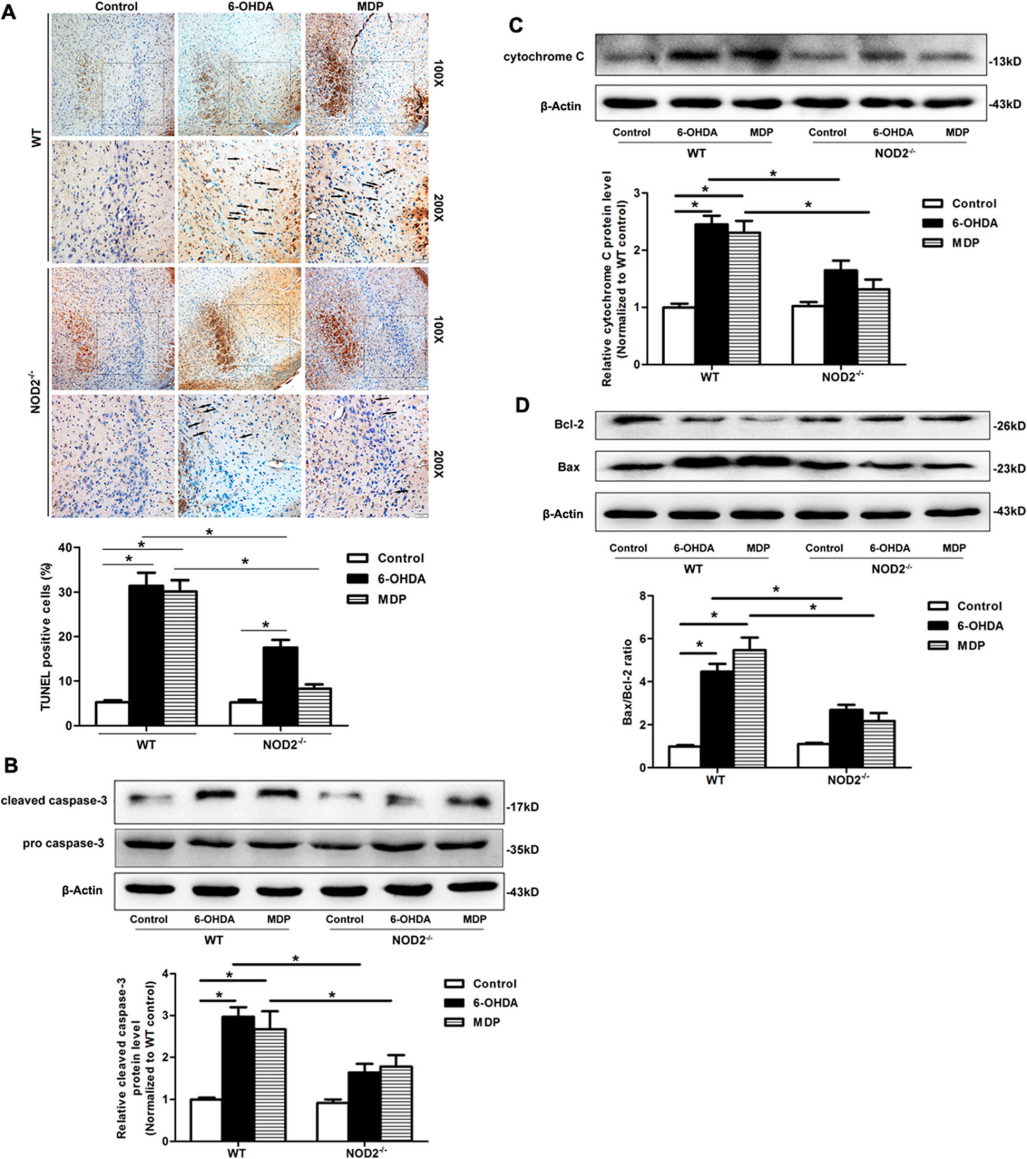
NOD2 deficiency attenuated the apoptosis in the substantia nigra (SN) induced by 6-OHDA. Wild-type (WT) and NOD2-deficient (NOD2^−/−^) mice were placed in a stereotaxic device under 1.5% pentobarbital sodium anesthesia and given 6-OHDA or saline alone into two different sites of the STR separately. **a** Representative images and quantifications of apoptosis based on TUNEL assay in the SN from WT or NOD2^−/−^ mice on 14 days after striatal infusion of 6-OHDA. Western blot analysis was used to measure the protein levels of cleaved caspase-3 and pro caspase-3 (**b**), cytochrome C (**c**), and Bcl-2 and Bax (**d**) in the SN on 14 days after 6-OHDA treatment. All data are presented as the mean ± SEM, *n* = 6 mice per group. *p < 0.05 vs. indicated group

To further investigate whether the degeneration of DA neurons mediated by NOD2 pathway is related to apoptosis, we performed TUNEL assay on the sections from the SN on 14 days after striatal infusion of 6-OHDA. The number of apoptotic cells from the SN in WT mice after the injection of 6-OHDA or MDP was significantly increased, while it was dramatically decreased in NOD2^−/−^ mice compared with WT mice with the same treatment (Fig. 4a). Of note, the number of apoptotic cells from MDP treatment group had no significant difference compared with NS group in NOD2^−/−^ mice. We further examined the protein level of Bax, Bcl-2, cytochrome C, and caspase-3, which are the proteins associated with apoptosis. As shown in Fig. 4b, c, 6-OHDA or MDP lesion induced the activation of caspase-3 and cytochrome C expression compared with the control group in WT mice, which was significantly inhibited by the deficiency of NOD2. In addition, the ratio of Bax and Bcl-2 protein in the SN in NOD2^−/−^ mice was also significantly decreased compared with WT mice (Fig. 4d).

### NOD2 signaling influenced inflammatory reaction in the SN and STR

**Fig. 5:**
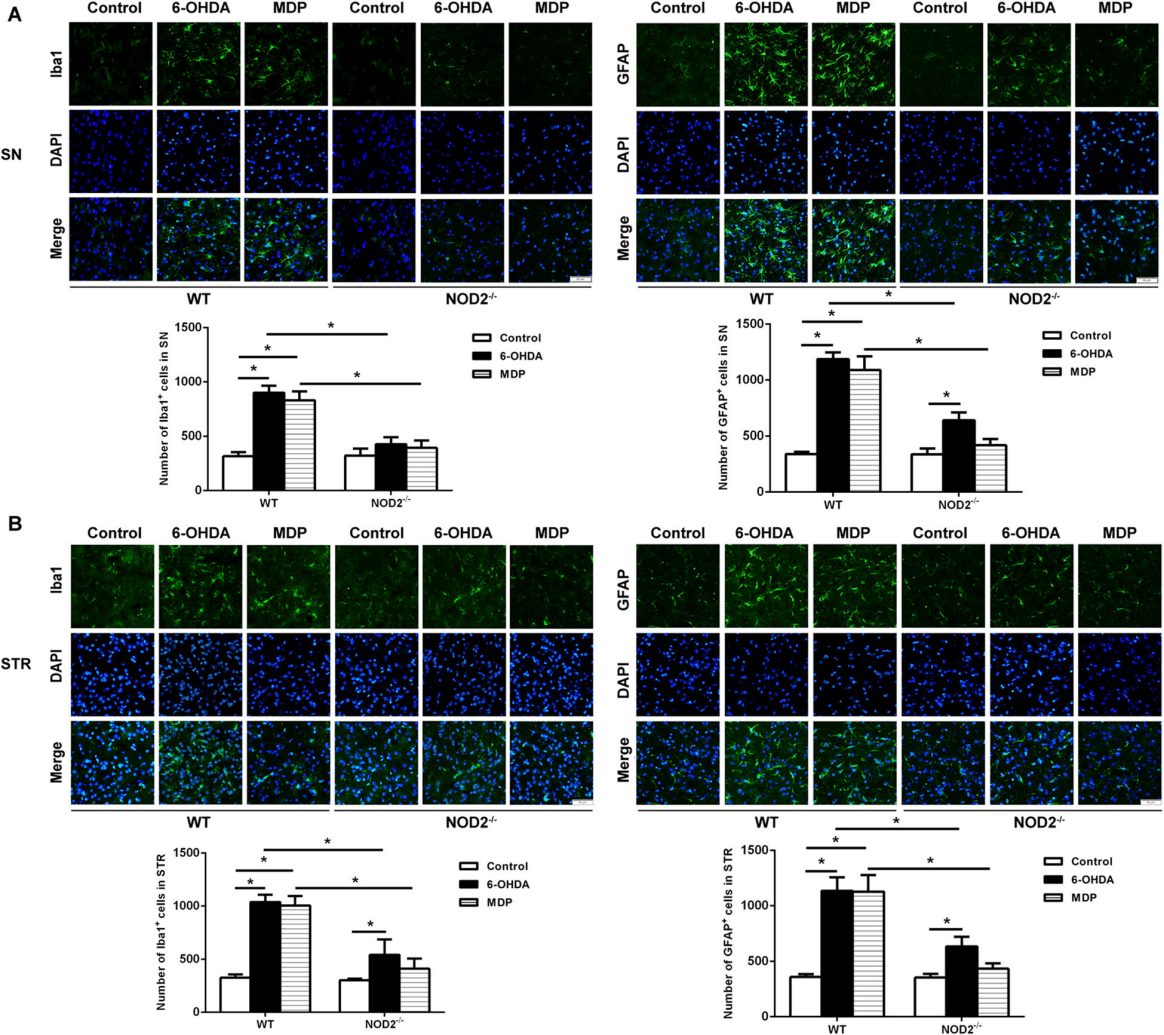
Microglial and astrocytic response within the substantia nigra (SN) (**a**) and striatum (STR) (**b**) in wild-type (WT) and NOD2-deficient (NOD2^−/−^) mice after striatal injection of 6-OHDA. The brains were isolated on 14 days after 6-OHDA injection and immunostained for Iba1 (green) and GFAP (green). Immunofluorescent staining indicated that 6-OHDA or MDP promoted microglia activation and astrocytic reaction, whereas NOD2 deficiency reduced the microglial and astrocytic response induced by 6-OHDA or MDP. The cell nuclei were counterstained with DAPI. Scale bars, 50 μm

To evaluate the neuroinflammatory reaction, we examined microglial activation by Iba1 immunostaining and astrocytic activation by GFAP immunostaining in the SN and STR of WT and NOD2^−/−^ mice at 14 days after the administration of 6-OHDA, MDP, or NS. As shown in Fig. 5, 6-OHDA or MDP injection induced microglial activation reflected by the presence of Iba-immunoreactive cells with rod-like morphology and increased GFAP immunoreactivity from the SN and STR in WT mice. However, both microglial and astrocytic activations were evidently attenuated in NOD2^−/−^ mice after 6-OHDA or MDP injection compared with WT mice.

**Fig. 6:**
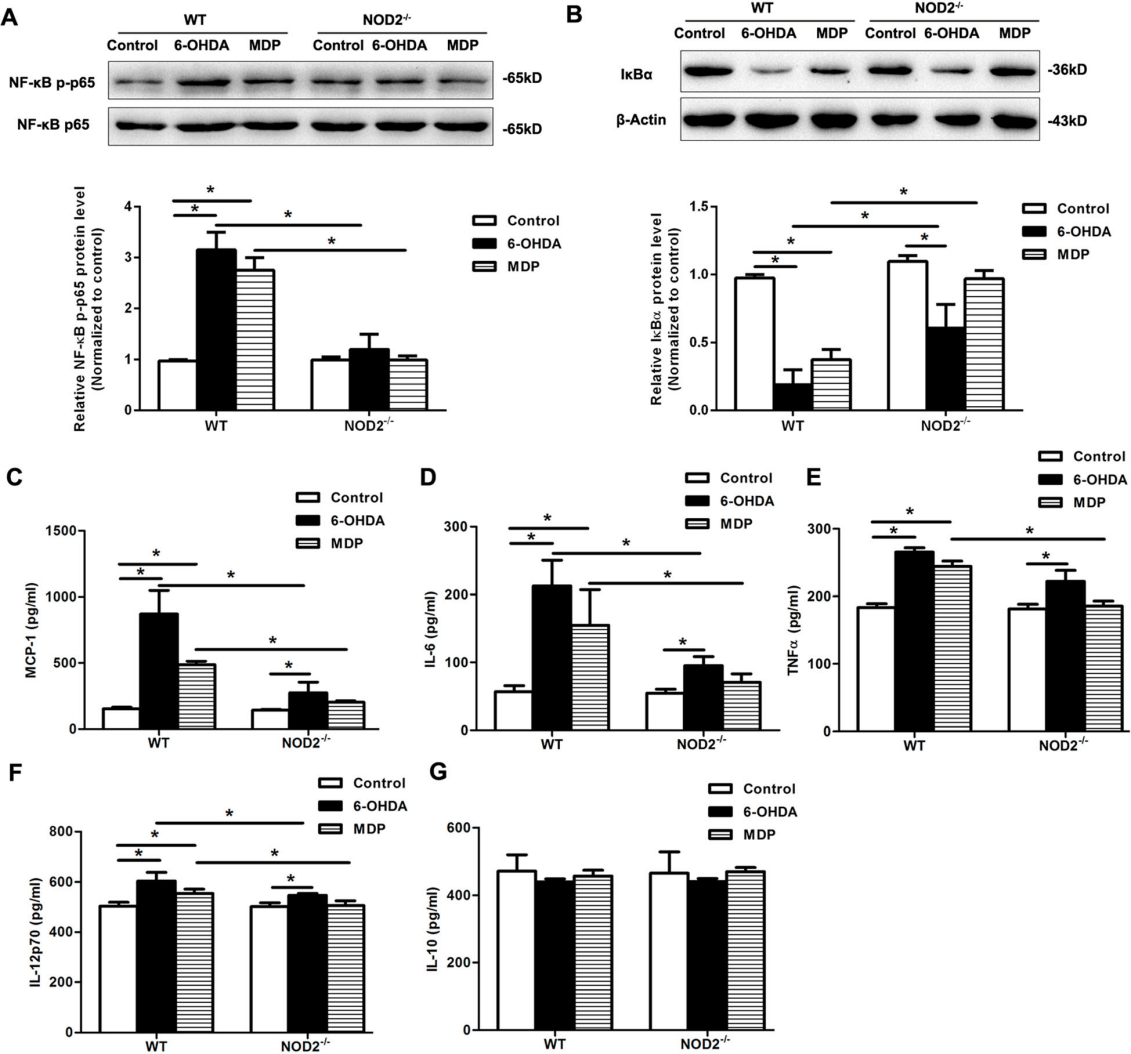
Effect of NOD2 signaling on inflammation in Parkinson’s disease mice induced by 6-OHDA. Wild-type (WT) and NOD2-deficient (NOD2^−/−^) mice were placed in a stereotaxic device under 1.5% pentobarbital sodium anesthesia and given 6-OHDA, muramyl dipeptide (MDP, an extrinsic ligand of NOD2), or saline alone into two different sites of the STR separately. Western blot analysis was used to measure NF-κB p-p65 (**a**) and IκBα (**b**) protein levels in the SN from WT and NOD2^−/−^ mice on 14 days after 6-OHDA or MDP treatment. The chemokine MCP-1 (**c**) and pro-inflammatory cytokine [IL-6 (**d**), TNF-α (**e**), IL-12p70 (**f**)] and anti-inflammatory cytokine IL-10 (**g**) on 14 days after striatal infusion of 6-OHDA or MDP in WT and NOD2^−/−^ mice were measured by cytometric bead array (CBA) multiplexed assay. All data are presented as the mean ± SEM, *n* = 6 mice per group.*p < 0.05 vs. indicated group

To profile the neuroinflammatory response to NOD2 in PD, we next measured the activity of NF-κB as well as the protein level of pro-inflammatory cytokines and chemokines at 14 days after the injection of 6-OHDA or MDP. As shown in Fig. 6a, b, the NF-κB pathway was markedly activated as evidenced by the phosphorylation of NF-κB subunit p65 and degradation in IκBα in the SN of WT mice in response 6-OHDA or MDP. The activation of NF-κB induced by 6-OHDA or MDP was significantly inhibited in NOD2^−/−^ mice. Furthermore, 6-OHDA or MDP treatment significantly increased the production of MCP-1, IL-6, TNF-α, and IL-12p70, which was markedly diminished in NOD2^−/−^ mice (Fig. 6c–f). Of note, the concentration of IL-10 was not altered after the lesion in either WT or NOD2^−/−^ mice (Fig. 6g).

### NADPH oxidase 2 (NOX2) was activated in PD mice induced by 6-OHDA

**Fig. 7:**
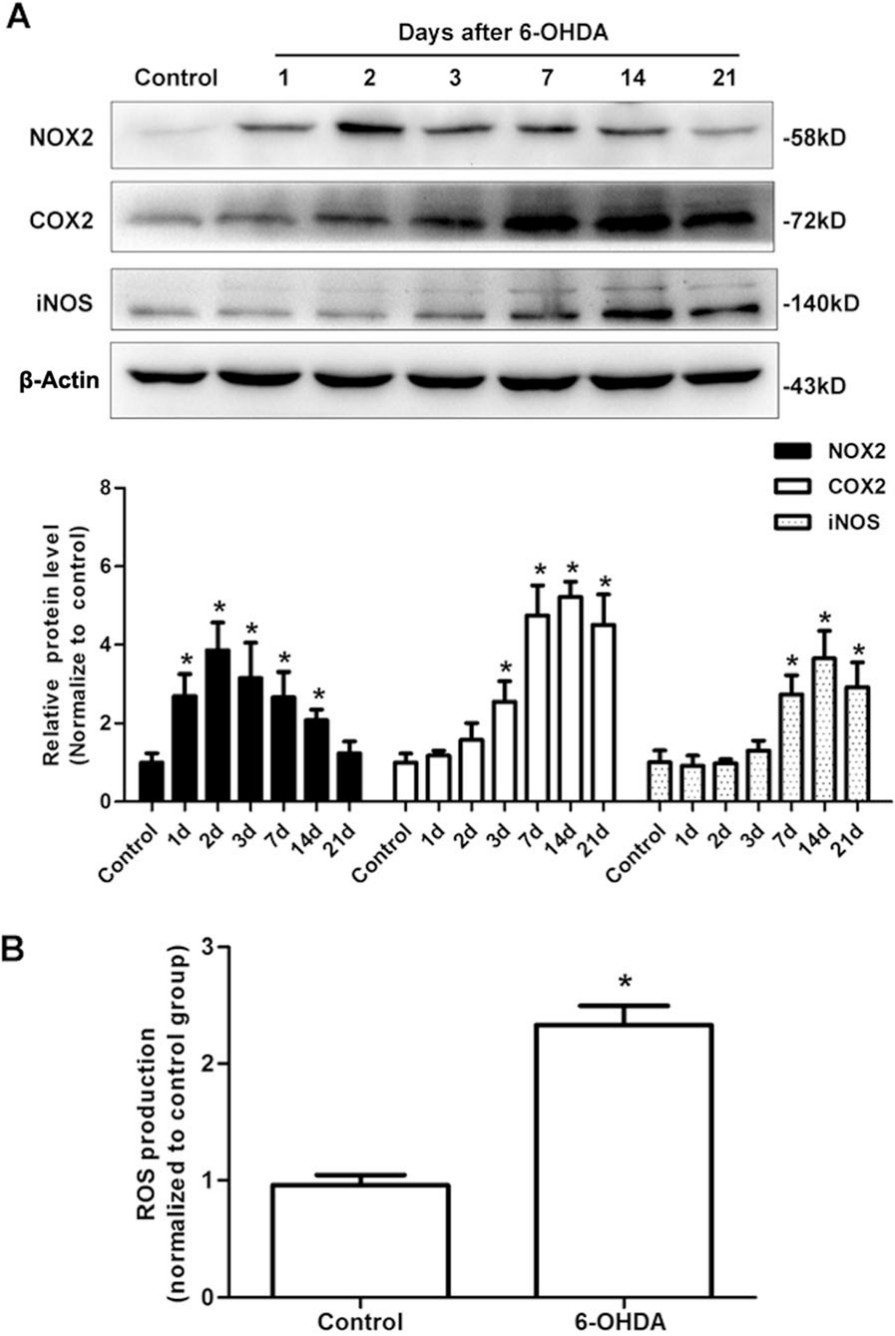
NADPH oxidase 2 (NOX2) was activated in Parkinson’s disease mice induced by 6-OHDA. Wild-type (WT) mice received a striatal injection of saline or 6-OHDA. NOX2, COX2, and iNOS expressions were measured by Western blot on 1 day, 2 days, 3 days, 7 days, 14 days, and 21 days after the injection (**a**). Seven days after striatal injection of 6-OHDA, the SN was collected for the measurement of reactive oxygen species (ROS) levels (**b**). All data are presented as the mean ± SEM, *n* = 6 mice per group. *p < 0.05, compared with indicated group

In order to investigate how NOD2 is activated in PD, we examined the expression of NADPH oxidase subunit NOX2 (also known as gp91phox) and ROS production in the SN at indicated time points after the injection of 6-OHDA. NOX2 protein level was increased in a time-dependent manner with a peak expression at 2 days after 6-OHDA injections (Fig. 7a), and the ROS production was also dramatically increased at 7 days after 6-OHDA injection (Fig. 7b). Interestingly, the enhanced expressions of NOD2 (Fig. 1b), COX2, and iNOS (Fig. 7a) were also observed in the SN after 6-OHDA injections, but the peak times were later than that of NOX2, which were 7 days, 14 days, and 14 days, respectively.

### NOX2-derived ROS was associated with NOD2 signaling in microglia induced by 6-OHDA

**Fig. 8:**
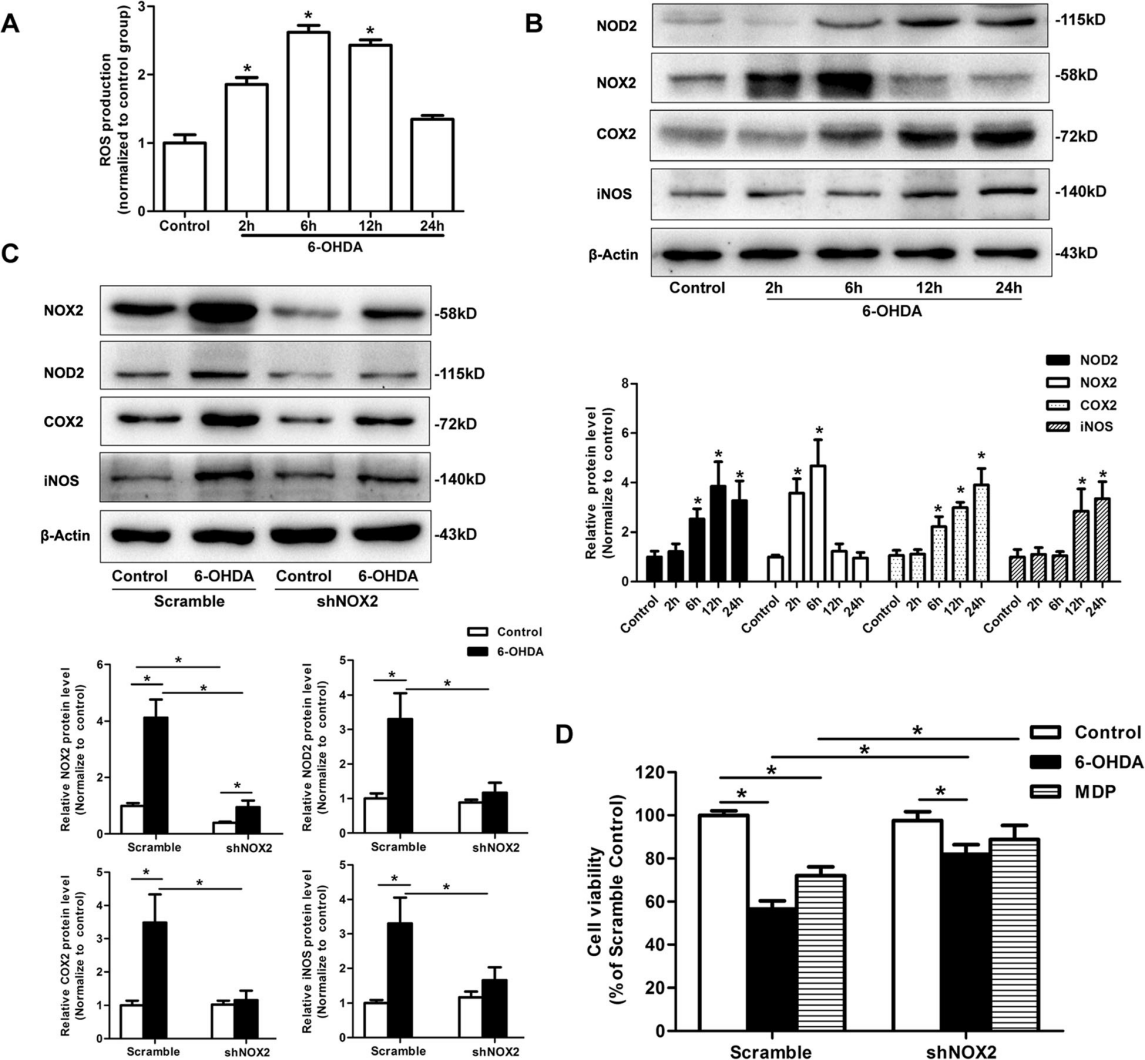
NADPH oxidase 2 (NOX2)-derived reactive oxygen species (ROS) was associated with NOD2 signaling in microglia induced by 6-OHDA. Reactive oxygen species (ROS) production (**a**) and NOD2, NOX2, COX2 and iNOS expressions (**b**) were induced by 50 μM 6-OHDA time-dependently in microglial BV2 cells. **c** The NOX2 silence inhibited the expression of NOD2, COX2, and iNOS in microglia induced by 6-OHDA. **d** Gene silencing of NOX2 in microglia attenuated the toxicity of conditioned medium from 6-OHDA or MDP-stimulated microglia to SH-SY5Y cells. SH-SY5Y cells were treated with conditioned medium from unstimulated microglia (control), 6-OHDA, or MDP-stimulated microglia for 24 h. Cell viability was assessed by the CCK-8 assay. Data are means ± SEM from three independent experiments. *p < 0.05, compared with indicated group

6-OHDA also significantly induced ROS production (Fig. 8s) and the expressions of NOX2, NOD2, COX2, and iNOS (Fig. 8b) in the microglial BV2 cells, but the peak time of NOX2 was earlier than others. Butovsky [35] recently reported the monocytes recruited to the CNS and microglia cell lines were clearly different from in vivo microglia. So we tested the effect of 6-OHDA on the NOD2 and NOX2 expression in primary cultured mouse microglia. In addition, we found 6-OHDA upregulated the expressions of NOD2 and NOX2 in primary microglia (Additional file 1: Figure S1). To elucidate the function of NOX2 in NOD2 activation, we next analyzed whether NOX2 knockdown could suppress NOD2 signaling in microglia injured by 6-OHDA. As shown in Fig. 8c, silencing of NOX2 displayed significantly diminished protein expression of NOD2, COX2, and iNOS in response to 6-OHDA compared to control microglia.

It is well known that overactivation of microglia leads to the release of neurotoxic cytokines that in turn may participate in the progression of neurodegeneration. To understand the role of NOX2 in NOD2-mediated neurotoxicity, the SH-SY5Y cells were cultured with BV2 CM collected from 6-OHDA or MDP-stimulated BV2 cells. The result showed SH-SY5Y cell viability was decreased after the exposure of 6-OHDA or MDP-treated BV2 CM for 24 h, indicating 6-OHDA or MDP-induced microglial overactivation may be harmful to the neurons. In contrast, NOX2 deficiency greatly improved the cell viability cultured with the 6-OHDA or MDP-CM (Fig. 8d).

### The NADPH oxidase inhibitor apocynin attenuated DA neuronal degeneration induced by 6-OHDA and MDP

**Fig. 9:**
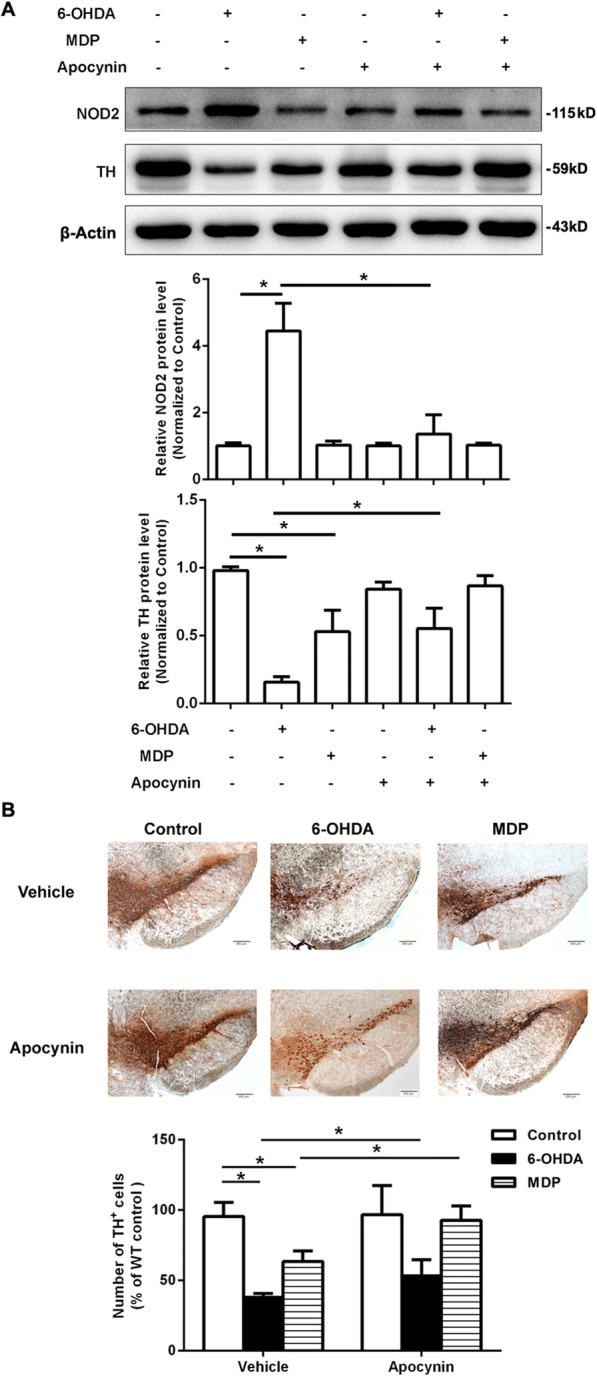
Wild-type (WT) mice were placed in a stereotaxic device under 1.5% pentobarbital sodium anesthesia and given 6-OHDA, muramyl dipeptide (MDP, an extrinsic ligand of NOD2), or saline alone into two different sites of the striatum separately. Apocynin was injected i.p. at a dose of 15 mg/kg for 14 days after 6-OHDA or MDP injections. **a** Apocynin improved the reduction of TH protein expression in the SN on 14 days after 6-OHDA or MDP injection. **b** The brain sections from the SN showing tyrosine hydroxylase (TH) immunoreactivity on 14 days after striatal infusion of 6-OHDA or MDP. Results are representative of six mice. *p < 0.05, compared with indicated group

The in vivo experiment revealed that apocynin treatment diminished the NOD2 upregulation induced by 6-OHDA (Fig. 9a). Furthermore, apocynin treatment inhibited the decrease in the number of TH^+^ neurons and TH protein levels in the SN of WT mice induced by 6-OHDA and MDP (Fig. 9a, b). These results suggested that NOX2-derived ROS regulated the NOD2-mediated DA degeneration in PD-like mice induced by 6-OHDA. On the other hand, we also explored the effect of NOD2 knockout on NOX2 in the 6-OHDA model. The result showed that the expression and activation of NOX2 induced by 6-OHDA or MDP were significantly inhibited in the NOD2^−/−^ mice (Additional file 2: Figure S2).

## Discussion

Here, we reported that NOD2 played a critical role in 6-OHDA-induced PD-like pathogenesis. We found that NOD2 was upregulated in the SN and STR in PD model mice induced by 6-OHDA. The striatal injection of NOD2 agonist MDP induced DA degeneration mice, while mice lacking NOD2 protected against the DA degeneration induced by 6-OHDA or MDP due to the attenuated inflammatory response. We further demonstrated NOX2-mediated oxidative stress linked NOD2 to DA neuronal degeneration induced by 6-OHDA.

Neuroinflammation is an important contributor to the neuronal loss in PD [2]. NOD2 has several important properties, which contribute to brain inflammation. It was identified as an important component in the generation of damaging central nervous system (CNS) inflammation following bacterial infection [36]. We recently reported NOD2 was involved in the inflammatory response after cerebral ischemia-reperfusion injury [33]. But the role and mechanism of NOD2 in PD are still unknown. Although there is no consensus on the genetic evidence supporting a pathogenic function of NOD2 in PD [28–30], we proved the NOD2 expression in the SN and striatum was upregulated in 6-OHDA-induced PD model mice in the present study. WT mice with the treatment of NOD2 agonist MDP displayed the injury of DA neurons, while NOD2^−/−^ mice were partially protected against 6-OHDA toxicity. These findings indicated that DA cell death in this paradigm is, at least to some extent, NOD2-dependent.

Microglia, the resident innate immune cells, are sensitive to even minor disturbances in CNS homeostasis and become readily activated during most neuropathological conditions [37]. Activated microglia is thought to promote neuronal damage, particularly in neurodegenerative diseases, via the release of pro-inflammatory and neurotoxic factors. Several studies have provided evidence that exposure of microglia to PAMPs or DAMPs leads to the release of inflammatory and toxic molecules that contribute to neurodegeneration [38, 39]. The astrocytic response has also been reported to play an important role in the events leading to DA degeneration, a response which seemingly occurs following microgliosis [19]. We observed the obvious activation of microglia and astrocytes displayed in WT mice treated with 6-OHDA or MDP, while this activation was significantly attenuated by NOD2 deficiency. It has been shown that activated microglia can produce and release, in excess, a host of harmful compounds such as ROS, reactive nitrogen species, and pro-inflammatory cytokines. Evidence has also suggested the activities of nuclear factor kappa B (NF-κB) are the potential mechanisms mediating activated microglia-associated DA degeneration [40]. NF-κB activation drives the expression of inflammatory cytokines in PD. Our results also showed that the activation of NF-κB, as evidenced by IκBα degradation and p65 phosphorylation, was significantly inhibited in NOD2^−/−^ mice compared with WT mice treated with 6-OHDA or MDP. Congruently, inflammatory cytokines including MCP-1, IL-6, IL-12, and TNFα were produced in less amount in NOD2^−/−^ mice. These findings for the first time demonstrated that NOD2 is one of the critical components in a signal transduction pathway that links 6-OHDA toxicity to inflammation in PD.

Apoptosis plays a fundamental role in the regulation of cellular homeostasis and is involved in DA neuronal loss in PD [41, 42]. NOD2 has been causally linked to the pathogenesis of apoptosis [43]. In our study, treatment with 6-OHDA or MDP resulted in a significant increase in apoptosis of DA neurons in the SN from WT mice, while NOD2 deficiency reduced the apoptosis induced by 6-OHDA or MDP. Caspase-3, a member of the cysteine proteases, is implicated in the apoptosis. Activation of caspase-3 is associated with a series of signal transduction cascades including cytochrome C, Bcl-2, and Bax proteins. Bcl-2 can form a heterodimer with Bax, thereby preventing Bax homodimerization and causing an alteration in mitochondrial permeability. The change in the permeability of mitochondria leads to cytochrome C release, formation of the apoptosome, and the subsequent activation of caspase-3 [44]. Furthermore, the activation of caspase-3-like proteases has been shown to be involved in the pro-apoptotic function of neurotoxins in MPTP and 6-OHDA models [45]. MPTP-induced dopaminergic neurodegeneration was paralleled with the upregulation of Bax and downregulation of Bcl-2 in the SN, and mutant mice lacking Bax are more resistant to MPTP than their WT littermates [46]. The present study revealed that the ratio of Bax/Bcl-2, cytochrome C, and caspase-3 activation was markedly inhibited in NOD2^−/−^ mice compared with WT mice when treated with 6-OHDA or MDP, suggesting the pro-apoptotic effect of NOD2 in PD. It is conceivable that NOD2 may exert its pro-apoptotic effects in PD by regulating cytochrome C, Bax, and Bcl-2, thus promoted the activation of caspase-3.

A particularly intriguing question is how NOD2 was upregulated in 6-OHDA induced PD model mice. Recent studies have indicated that activation of the immune system due to the disturbances in the redox state of cells seems to contribute to DA damage [47], and NOX2-derived ROS are central to oxidative stress in PD [48, 49]. Seven NOX isoforms have been identified, namely, NOX1, NOX2, NOX3, NOX4, NOX5, and dual oxidase 1 and 2 (DUOX1 and DUOX2). Structurally, all members of the NOX family contain a multisubunit structure, with catalytic flavin-binding NOX subunits and a number of regulatory subunits. Among them, NOX2 is the most important subtype for mediating PD injury. NOX2 is intensely expressed in microglial cells and has been demonstrated to be involved in the degeneration of DA neurons induced by MPTP or 6-OHDA [48, 50, 51]. Our results showed NOD2 was also expressed in microglia and upregulated by 6-OHDA. Moreover, gene silencing of NOX2 suppressed the expression of NOD2 and the subsequent inflammatory response in microglia. We further confirmed that apocynin, a NADPH oxidase inhibitor, inhibited NOD2 upregulation and prevented DA degeneration in mice induced by 6-OHDA or MDP. These results suggested that NOX2-derived ROS contributed to the upregulation of NOD2 and the following inflammatory responses induced by 6-OHDA. In line with these findings, Shang et al. recently proved that enhanced NOD2 expression caused by high glucose in mesangial cells is regulated via the translocation of HuR, which is dependent on the NOX4-mediated redox signaling pathways [52]. Of course, the detailed mechanisms of NOD2 regulated by NOX2 in PD needs to be further clarified. Moreover, we previously demonstrated NOX2-derived ROS mediated NOD2-dependent inflammatory responses in cerebral ischemia-reperfusion [33]. Interestingly, we also found that the expression and activation of NOX2 induced by 6-OHDA or MDP were significantly inhibited in the NOD2^−/−^ mice compared to WT mice (Additional file 2: Figure S2). Overall, the results indicate that NOD2 and NOX2 form a positive circuit and promote DA degeneration in 6-OHDA-induced PD-like pathology in mouse models.

## Conclusion

In summary, this study for the first time proved that NOD2 was a novel innate immune signaling regulation in the midbrain inflammatory response and provided direct evidence that NOX2-mediated oxidative stress linked NOD2 to DA degeneration induced by 6-OHDA. Pharmacological targeting of NOD2-mediated signaling pathways at multiple levels may provide a novel approach for the treatment of PD.

## Additional Files

Additional file 1: **Figure S1.** 6-OHDA upregulated the expression of NOD2 and NOX2 in primary microglia. (TIF 2105 kb)

Additional file 2: **Figure S2.** NOD2 deficiency reduced the expression of NOX2 and the production of reactive oxygen species (ROS) induced by 6-OHDA or MDP. (TIF 1274 kb)

## Abbreviations

6-OHDA: 6-Hydroxydopamine
DA: Dopaminergic
DOPAC: Dihydroxyphenylacetic acid
HVA: Homovanillic acid
MDP: Muramyl dipeptide
MPTP: 1-Methyl-4-phenyl-1,2,3,6-tetrahydropyridine
NLRs: NOD-like receptors
NOD2: Nucleotide-binding oligomerization domain-containing protein 2
NOX2: NADPH oxidase 2
PD: Parkinson’s disease
ROS: Reactive oxygen species
SN: Substantia nigra
TLRs: Toll-like receptors
