# Supplementary figures and images for: NOD2 promotes dopaminergic degeneration regulated by NADPH oxidase 2 in 6-hydroxydopamine model of Parkinson’s disease
Source: J Neuroinflammation. 2018 Aug 29;15:243. doi: 10.1186/s12974-018-1289-z (PMC6116377; doi:10.1186/s12974-018-1289-z)

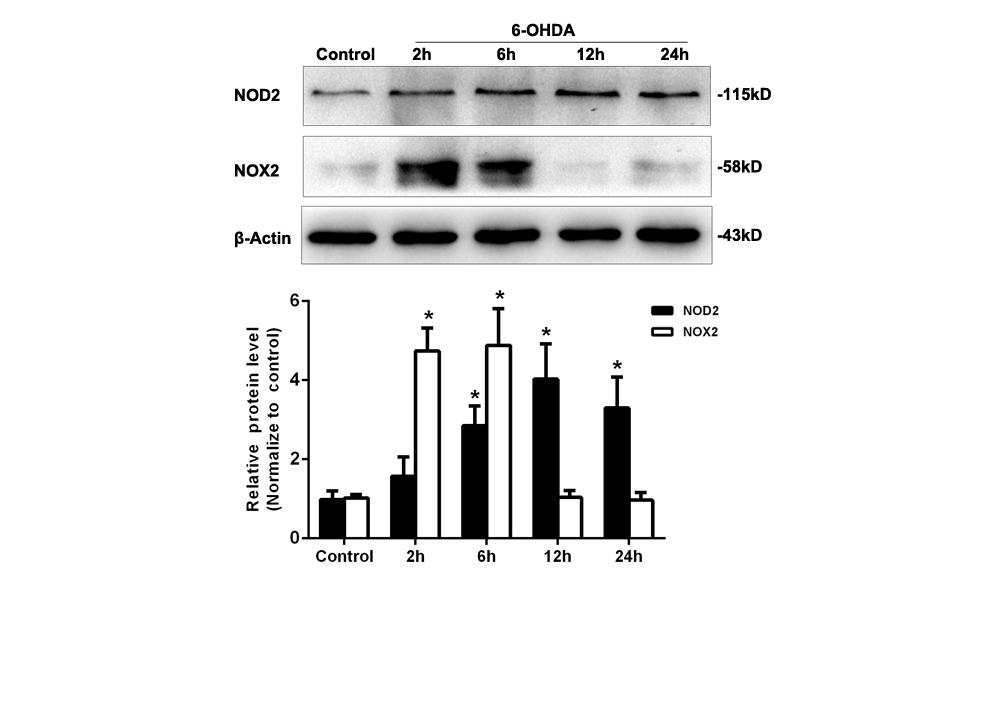

Supplement: Supplementary file 1 — Figure S1. 6-OHDA upregulated the expression of NOD2 and NOX2 in primary microglia. (TIF 2105 kb) [file 12974_2018_1289_MOESM1_ESM.tif]

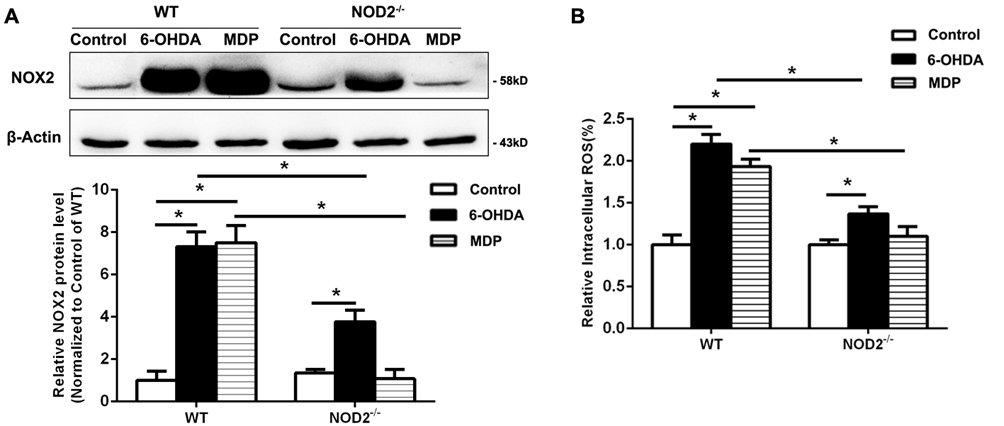

Supplement: Supplementary file 2 — Figure S2. NOD2 deficiency reduced the expression of NOX2 and the production of reactive oxygen species (ROS) induced by 6-OHDA or MDP. (TIF 1274 kb) [file 12974_2018_1289_MOESM2_ESM.tif]
